# A study protocol for a randomized controlled trial to assess the efficacy of Baduanjin exercise on older adults with sarcopenia in China

**DOI:** 10.1186/s12906-022-03778-9

**Published:** 2022-11-18

**Authors:** Shengnan Yue, Jing Zhang, Jiaxin Li, Yanwei Hao, Shaofeng Wang, Tianyu Liu, Wen Zhong, Chongli Chen, Fei Wang, Bin Li

**Affiliations:** 1 Department of Geriatrics, Hospital of Chengdu University of Traditional Chinese Medicine, Chengdu, 610072 Sichuan China; 2 School of Clinical Medicine, Chengdu University of Traditional Chinese Medicine, Chengdu, 610075 Sichuan China; 3 School of Physical Education and Health, Chengdu University of Traditional Chinese Medicine, Chengdu, 611137 Sichuan China

**Keywords:** Baduanjin, Sarcopenia, Older adults, Physical function, Randomized controlled trial

## Abstract

**Background:**

Accompanied by the decline of physiological functions, the decrease of physical activity, and comorbidities, older adults are susceptible to sarcopenia because of accelerated loss of muscle mass. Resistance training is recommended by different clinical practice guidelines. However, most older adults have difficulty in taking recommended high-load resistance training programs, and there are limited exercise options form them. Baduanjin, a traditional Chinese mind-body exercise with relatively low intensity and simple movements, has the potential benefits of improving physical functions and may be feasible in treating sarcopenia and preventing its adverse health outcomes in older patients. With the emergence of the concept of gut-muscle axis, this study aims to determine the efficacy of Baduanjin exercise on Chinese older adults with sarcopenia and explore its underlying mechanism.

**Methods:**

This is a 24-week, assessor-blinded, randomized controlled trial. Individuals aged 60 to 84 years old will be screened for sarcopenia. 90 participants with sarcopenia will be enrolled and randomly assigned (1:1) into the Baduanjin exercise or resistance training group, and 20 participants without sarcopenia will be set as the non-sarcopenia control group. The primary outcome is the scores of Short Physical Performance Battery. The secondary outcomes are body composition, handgrip strength, walking speed, global cognitive function, and incidence of falls. These outcomes will be assessed at baseline, the 12th week and the 24th week. While stool samples from participants will be collected at baseline and the 24th week for analyzing the abundance of gut microbiome. Data will be analyzed in an intention-to-treat protocol.

**Discussion:**

The results of this study will determine whether Baduanjin exercise can be an alternative non-pharmacological approach for older adults with sarcopenia. If they can show positive significance, it will promote Baduanjin exercise in clinical practice among these patients and inform further research involving exercise interventions on the optimal types, timing, and intensity to ameliorate sarcopenia for elderly people.

**Trial registration:**

Chinese Clinical Trial Registry; Registration number: ChiCTR2100051871; Prospectively registered on October 8th, 2021.

**Supplementary Information:**

The online version contains supplementary material available at 10.1186/s12906-022-03778-9.

## Background

Although life expectancy is increasing worldwide, it does not always translate to health span. Older adults are vulnerable to sarcopenia, a progressive skeletal muscle disorder involving the loss of muscle mass and function [1]. Report from the Asian Working Group for Sarcopenia (AWGS) in 2017 indicated that the prevalence of sarcopenia in people aged over 40 years ranged from 5.5 to 25.7% in Asian countries [2]. Attention should be paid to sarcopenia, because the link between low muscle strength and adverse health outcomes, like falls and functional decline, is long established [1, 3]. Additionally, these consequences can bring about impaired quality of life and increased health-care costs to both individuals and societies [1].

Pathophysiological mechanisms and pathways of sarcopenia are complex involving aging, imbalance between adipose tissue and skeletal muscle, altered mitochondrial integrity, endocrine abnormalities [1, 4]. Although its pathophysiology has not yet been fully understood, and there are no approved drugs for the treatment of sarcopenia currently, evidence-based clinical practice guidelines strongly recommend physical activity as the primary treatment of it [1, 5, 6]. Evidence for the benefits of resistance exercise in improving muscle mass, strength, and physical function is compelling [1, 6, 7]. However, most older adults may have difficulty in meeting the recommendations from the American College of Sports Medicine and the American Heart Association, suggesting them engage in high-load resistance training programs [8, 9]. Thus, it is necessary to find a safe and feasible exercise program for older people with sarcopenia to improve skeletal muscle mass and strength and prevent them from falls or injuries.

Baduanjin exercise, also called Eight-Section Brocades, is one of the martial arts to prevent disease and keep fit, using soft stretching movements. Like another Chinese Qigong exercise, Tai Chi, it is also a form of meditative exercise for maximizing both mental and physical health [10]. Because of its emphasis on the integration of body and mind, this exercise modality can convey the concept of *intervening before sick and treat before change* in Chinese medicine and has an advantage in facilitating holistic rehabilitation of an individual. Compared with Tai Chi, however, Baduanjin does not require specific equipment or venue or too many positions of body balance and can be easily learned. It is safe and suitable for older adults and populations with cognitive impairments or chronic diseases who have relatively low tolerance to physical exercise intensity and temperature change [10–12]. For elderly individuals, a series of simple movements may give them enjoyment and build their confidence to make efforts during exercise. Since emerging studies demonstrate that Baduanjin show positive effects on physical function, balance, gait, falls, pain, quality of life, and frailty status among elderly individuals and neurodegenerative patients [13–15]. Studies performed among elderly patients with sarcopenia are relatively limited. Thus, it is reasonable that Baduanjin can potentially improve the health-related parameters in older adults with sarcopenia.

With the growing interests in studying the role of gut microbiome in diseases, a hypothesis of gut-muscle axis has been raised, pre-clinical and clinical evidence indicate that altering the gut microbiome can affect muscle phenotypes including muscle mass and physical performance, and probiotics supplementation can restore age-related muscle loss [16–19]. The gut microbiota can provide nutrients for muscle synthesis or act as a regulator of muscle metabolism associated with the pathogenesis of sarcopenia [18]. A study observed that the composition and function of gut microbiome have changed in young men who exercise regularly after a period of cessation of exercise, suggesting that gut microbiome will alter in response to exercise habits change [20]. Meanwhile, physical activity and lifestyle tend to change with age, which may attribute to gut dysbiosis among elderly people [18, 21]. Therefore, we hypothesize that Baduanjin exercise is beneficial for older adults with sarcopenia, and it may work via regulating gut microbiome.

To investigate the effects and underlying mechanisms of Baduanjin exercise on sarcopenia among older adults, we will conduct a randomized controlled trial. The main objective of this study is to determine the effects of Baduanjin exercise on the physical functions of older adults with sarcopenia. In addition, 16SrRNA analysis will also be applied to explore how Baduanjin exercise influences the gut microbiome of the elderly patients with sarcopenia.

## Methods/design

### Aim

To clinically evaluate the effects of regular Baduanjin exercise prescription on senile sarcopenia patients and explore its underlying mechanism related to gut microbiota. This will enable determination of potential intervention strategies for older adults with sarcopenia.

### Design and setting

This is an assessor-blinded, randomized controlled trial in which the older adults with sarcopenia living in Chengdu, China, will be randomly allocated in a 1:1 ratio into two arms: 1) Baduanjin exercise (BE), and 2) resistance training (RT). A total of 90 eligible patients with sarcopenia will be enrolled in the study. Set 20 non-sarcopenia older individuals randomly selected as the non-sarcopenia control (NC) group.

**Fig. 1:**
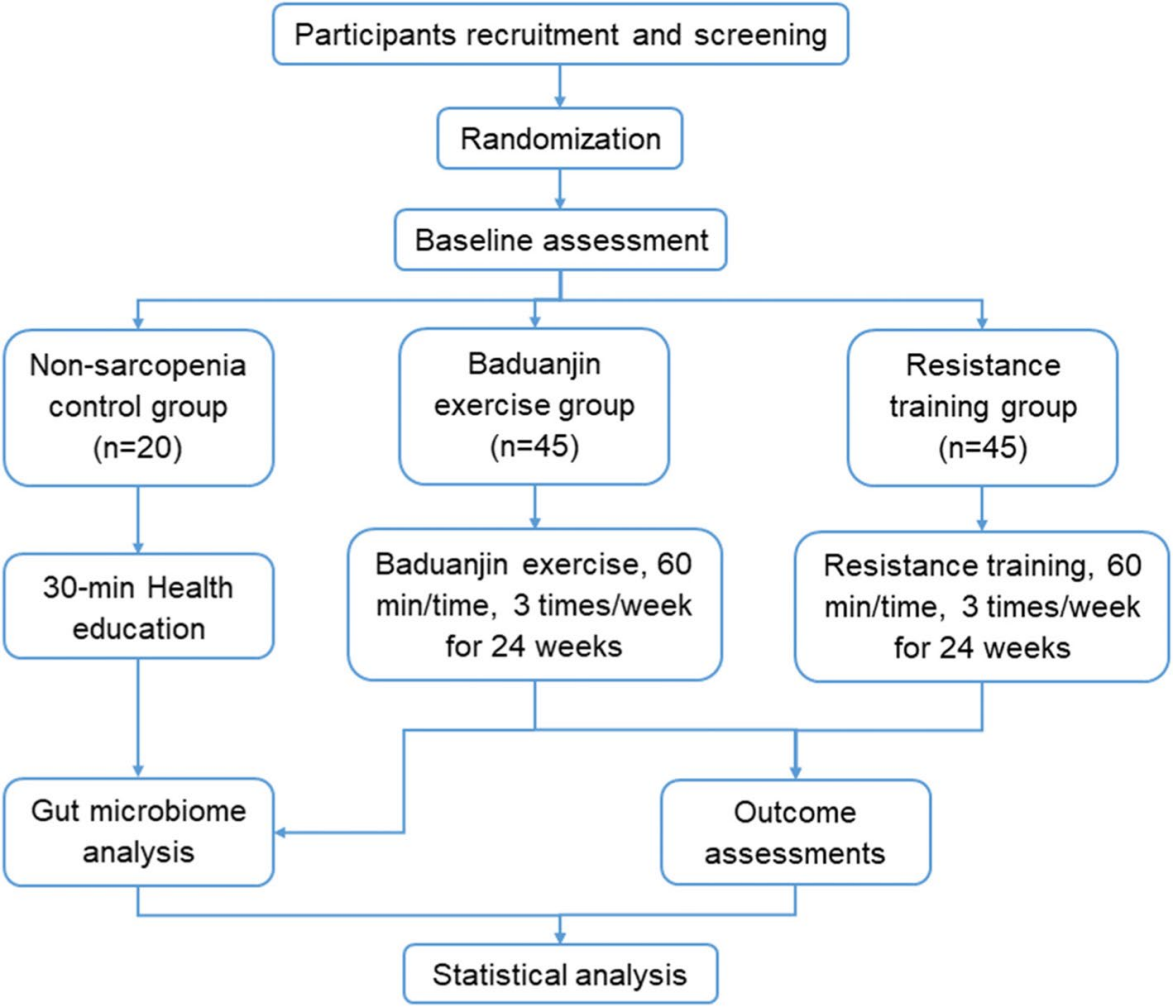
Flow chart of the trial

Baseline, midpoint and endpoint and trial assessments will be conducted. The flow chart is shown in Fig. 1. The current protocol (version 2.0, Dec. 3rd, 2021) met the principles of the Declararion of Helsinki, and was in accordance with the standard Protocol Items: Recommendations for Interventional Trials (SPIRIT) 2013 checklist is shown in Additional file 1. The recruitment started in February 2022 at Hospital of Chengdu University of Traditional Chinese Medicine.

### Characteristics of participants

#### Diagnostic criteria

According to the criteria proposed by the Asian Working Group for Sarcopenia (AWGS), patients with sarcopenia should have: (1) Based on bioelectrical impedance analysis (BIA), skeletal muscle mass index (SMI) in male ≤7.0 kg/m^2^, female ≤5.7 kg/m^2^; and (2) Handgrip strength (HS) in male ≤26 kg, female ≤18 kg; or (3) Gait speed < 0.8 m/s.

#### Inclusion criteria

(1) Patients with sarcopenia should:

1. be 60–84 years old and meet the diagnostic criteria for sarcopenia;
2. be able to cooperate with the completion of body composition analysis, grip strength measurement, 6 m walking time measurement and questionnaires;
3. be able to walk independently, with or without assistive devices;
4. be able to safely complete the exercise recommended by physicians;
5. agree to participate in this study, accept and implement the intervention program, and sign the informed consent.

(2) Participants who will be assigned to non-sarcopenia control group should:

1. be 60–84 years old;
2. not be diagnosed with sarcopenia.

#### Exclusion criteria

1. Patients whose 6 m walking speed < 1.0 m/s, or time for 5 sit-up times ≥12 s, or score of SPPB ≤9 points.
2. Patients with diseases which have a seriously negative influence on muscle and bone metabolism, such as malignant tumor, cerebrovascular accident, severe liver disease, severe kidney disease, severe diabetes mellitus, thyroid disease, parathyroid disease, etc.
3. Patients who are taking drugs that have a seriously negative influence on muscle and bone metabolism, such as glucocorticoids, androgens, estrogens, thyroid hormones, etc.
4. Patients who have a habit of exercising: they take at least 15 minutes of high-intensity physical activity daily, or muscle strength training at least twice a week for 3 months before enrollment.
5. Patients have severe cognitive impairment, whose score of mini-mental state examination (MMSE) is less than 20 points.
6. As for participants who can be candidates to offer their stool sample for gut microbiome analysis, when they:
  1. have gastrointestinal diseases or related surgical history;
  2. take drugs like antibiotics, gastrointestinal motility drugs, microecological regulators 2 months, etc., before stool collection.

#### Suspension criteria

1. During the study period, subjects are unwilling to continue follow-up visit and ask to stop this trial.
2. Loss of follow-up.
3. During the study period, if subjects have some comorbidities, complications, or special physiological changes, they may not be suitable to continue to receive interventions or participate in the trial.
4. During the study period, if a subject’s condition is aggravating and has a possibility of suffering a critical event, his or her attending physician will examine the subject and decide that the trial should be stopped.

#### Elimination criteria

1. After the start of the study, it is found that the subjects do not meet the inclusion criteria.
2. During the study period, the subjects refuse to take examinations or evaluations.
3. The subjects have poor compliance, as their cumulative time of exercise is less than 80% of the amount to be completed or more than 120% of that, or they are taking drugs that can affect muscle and bone metabolism during the period.

### Recruitment

Participants will be recruited from Hospital of Chengdu University of TCM through a combination of online and offline efforts including using the Internet, posting posters, and holding health education lectures. Potentially eligible individuals will first complete a screening by physicians in the department of geriatrics of Hospital of Chengdu University of TCM to determine their eligibility according to the inclusion and exclusion criteria. The eligible ones who are interested in taking part in the study will have an informed discussion with trained research assistants. The research assistants will obtain written informed consent from individuals willing to participate before starting the baseline assessment.

### Randomization, allocation concealment and blinding

A statistical analyst generated a random allocation sequence by statistical software SAS 9.4 (SAS, USA). After the baseline assessment, the eligible patients will be numbered in the order of inclusion and randomly assigned in a 1:1 ratio to the BE group or the RT group. And the allocation sequence has been kept by an independent research assistant who is not involved in recruitment, evaluation, or intervention of the participants. As for eligible non-sarcopenia participants, they will also be numbered from 1 to 20 in the sequence of inclusion.

Because this is a behavioral intervention, participants, exercise coaches and intervention supervisors will not be blinded to the assigned treatment, but outcome evaluators and data statisticians will be blinded to group allocation, i.e., letters A and B will be used instead of group names in case report forms. And the blindness will be revealed when all statistical analyses are completed.

### Interventions

Both intervention groups will receive 30-minute health education monthly about sarcopenia in old people, including its risk factors, adverse consequences, and preventive measures, such as balanced diet and lifestyle recommendations.

In two intervention groups, participants will receive three 60-minute weekly exercise sessions for 24 weeks. And each session will consist of a 15-minute warm-up activity (like trunk rotation, light walking, arm and leg extension, neck and ankle circles, so as to prevent injury during exercise), 30-minute core exercise (i.e. Baduanjin exercise or resistance training), and a 15-minute cool-down activity (to eliminate fatigue and restore physical finess), with a 30-second rest interval between exercises. Besides, both groups should complete three pre-intervention lessons with the corresponding sets of exercises to familiarize themselves with the training sessions.

Due to different enrolling time points and dwellings of participants, intervention classes may vary in size, with 8 to 20 participants. Professional coaches from the Chengdu University of TCM with at least 5-year teaching experience will be employed to guide participants through workouts.

Exercise intensity will be assessed according to a percentage of maximum heart rate (HR) monitored by a heart rate monitor (MIO, USA) during the whole exercise process. The average HR output after exercises is the measured HR of exercises. The calculation formula is as follows:

$$Percentage\ of\ maximum\ HR=\frac{measured\ HR}{220- age}\times 100\%$$

The intensity of exercise should be moderate to above-average, that is, 60 to 80% of maximum HR.

The researchers will record the attendance of each participant in each training session. If a participant misses a scheduled training session, he or she will be advised to complete the session within the training week. Besides, participants whose exercise time is less than 20 minutes will also be regarded as absent from that session. After the whole intervention period, exercise attendance will be calculated by the following formula:

$$Attendance\ rate\ \left(\%\right)=\frac{number\ of\ present\ sessions}{number\ of\ planned\ sessions}\times 100\%$$

#### Resistance training group

Considering physical condition of elderly people, the resistance training protocol will be specially designed for them so that it is low- to moderate-intensity and safe. And it will consist of 3 sets of exercises, including 30-sencond still wall squats, 15 repetitions of wall push-ups, 10 repetitions of lunge squats, 30-second backhand plank, and 15 repetitions of wall calf raises, respectively. There will be a 20-second rest interval between sets.

#### Baduanjin exercise group

The training scheme of Baduanjin exercise originates from the “Health Qigong Baduanjin Standard” enacted by the State Sports General Administration in 2006 [22]. The whole set of Baduanjin exercise consists of 10 postures [23], including: 1) preparation poseture, 2) prop up the sky by two hands, 3) draw a bow on both sides, 4) raise single arm up side by side, 5) look back and forwards, 6) shack head and buttocks, 7) pull toes with both hands, 8) clench one’s fist and glare, 9) rise and fall on tiptoe, and 10) ending posture. At each session, participants in the BE group should perform 2 to 3 sets of Baduanjin exercises, and they will have a 20-second rest interval between sets.

#### Non-sarcopenia control group

Participants without sarcopenia will be asked to take baseline assessment and offer their stool samples for gut microbiota analysis, and they will not receive any interventions but health education for once.

### Baseline and outcome assessment

Basic characteristics will be measured at baseline. Except gut microbiome analysis, all outcomes will be assessed at baseline, the 12th week (midpoint), and the 24th week (the end of the intervention). Stool samples of participants from the NC group will be collected at baseline for gut microbiome analysis, and those of eligible participants from the BE group and RT group will be collected at baseline and the 24th week. Additionally, these samples will be collected, tested, and recorded by the same group of researchers.

The enrollment process, interventions, and assessments are presented in Table 1.

**Table 1:** Schedule of trial procedures

Period	Screening	Intervention period		
Time point	0w	0w	12w	24w
**Enrollment**				
Eligibility screening	**×**			
Informed consent	**×**			
**Allocation**		**×**		
**Intervention**				
Non-sarcopenia control		**×**		
Baduanjin exercise		**×**	**×**	**×**
Resistance training		**×**	**×**	**×**
**Assessments**				
Physical function		**×**	**×**	**×**
Body composition		**×**	**×**	**×**
Handgrip strength		**×**	**×**	**×**
6 m walking test		**×**	**×**	**×**
Cognitive function		**×**	**×**	**×**
Incidence of falls		**×**	**×**	**×**
Gut microbiome		**×**		**×**

#### Baseline characteristics

At enrollment, participants’ demographic information (age, sex, race/ethnicity, living arrangements, education), medical conditions, meditations, vital signs, fall-related information (number of admissions and falls in the last year), handedness, sleeping habits, smoking and alcohol history, and physical activity will be collected by physicians using self-designed questionnaire.

#### Primary outcome

The primary outcome is scores of SPPB, a standardized method for assessing physical function of older adults, which measured repeated chair stands, 3 increasingly challenging standing balance tasks, and a 4-m speed walk. Scores on the 3 tasks are combined to create an overall performance score of 0 (worst) to 12 (best), with higher values indicating better physical function.

#### Secondary outcomes

1. Body composition involving body weight, body water, fat mass, fat percentage, lean body weight, body mass index (BMI), and segmental muscle mass of trunk, upper and lower limbs, respectively, will be measured using Body Composition Analyzer (DBA-510, Donghuayuan, China), a bioelectrical impedance analysis.
2. Handgrip strength will be measured via a JAMAR electronic grip strength metre (Yuyan, China) to estimate upper limb muscle strength. Participants should stand up straight with upper limbs close to the body trunk, keep the elbow flexed at 90° and the forearm supinated. Then participants will be asked to “squeeze as hard as [they] can”. Handgrip strength will be measured twice sequentially on each side and the highest recording of the four measurements will be used for analysis.
3. 6-m walk test will be applied to evaluate participants’ muscle performance. It requires them to walk at their daily speed for 6 m. Walking speed will be calculated by the recorded time it takes to walk 6 m. The test will be performed twice, and the average walking time and speed will be recorded for analysis.
4. Global cognitive function will be measured using 30-item Montreal Cognitive Assessment (MoCA, Changsha version), which is a cognition screening instrument created and validated to evaluate cognitive function of multiple domains and more suitable for Chinese. It scores from 0 to 30, with higher score suggesting better cognitive function.
5. Incidence of falls will be ascertained on a monthly basis. Participants will be asked to use a “fall calendar” diary to record any fall event (defined as “land on the floor or the ground, or fall and hit objects like stairs or pieces of furniture, by accident”) and medical attention they may seek. Information on injurious falls will also be collected. Information on falls will be collected starting from the date of the 1st intervention class and continuing until the end of the intervention period or until a participant withdraws, dies, or loses to follow-up.
6. As for gut microbiome analysis, eligible participants will be asked to collect their stool samples in the morning with sterile stool boxes. The fresh samples will be quickly transported in a liquid nitrogen box to the laboratory for subpackaging. Then fecal genomic DNA will be extracted from these samples to get PCR amplification, and be sequenced for 16SrRNA analysis.

#### Safety measurements

The exercise intervention will be carried out under the supervision of researchers and trainers. The adverse events and serious adverse events during each training session will be recorded in detail and analyzed the relation between exercise intervention and these events. The serious adverse events will be reported to the principal investigator and the medical ethics committee. Medical services will be provided when necessary.

### Statistical analysis

#### Power calculation

Because of the loss of muscular mass and function, falls and fractures are common clinical manifestations and serious consequences in patients with sarcopenia. According to data reported in a previous trial on older adults at high risk of falling [24], take score of short physical performance battery (SPPB) as effect index, using two-sided test, with 90% power and 5% significance level, then the sample size of a single intervention group calculated through the corresponding formula is approximately equal to 36. And set the drop-out rate as 20%, 45 cases with sarcopenia will be needed in per group. Another 20 older adults without sarcopenia of the same age group will be randomly selected as the control group, that is, a total of 110 participants will be recruited in this study.

#### Data analyses

Following an intention-to-treat (ITT) protocol, those whose cumulative time of exercise reaches 80% of the amount to be completed and not higher than 120% of that will be classified into per protocol set (PPS). Categorical variables will be presented as percentages or frequencies. As for continuous variables, normally distributed data will be described by mean ± standard deviation, while non-normally distributed data will be described using median [interquartile range (IQR)]. Baseline demographic descriptors and outcome measures will be compared across groups by using analysis of variance for continuous variables and the *x*^2^ (or Fisher exact) test for categorical variables. Two-sided *P* values of less than 0.05 will be considered statistically significant. Analyses will be conducted using SPSS version 23.0 (IBM Corp) or GraphPad Prism version 7.0 (GraphPad Software).

For the gut microbiome analysis, sequenced data will be interpreted using the bioinformatics tools programmed in the Ion Reporter software (Thermo Fisher Scientific). QIIME algorithms will determine the bacterial diversity within a sample (α diversity) and among all the samples (β diversity). Additionally, multivariate data analysis with principal component analysis on the diversity indexes and comparisons of genus and species level data will be performed to reveal differences in the microbial composition between individuals with normal cognition and ones with aMCI. The Benjamini–Hochberg false discovery rate adjustment will be used to account for the number of taxa tested in each comparison.

#### Data management

The research data will be collected by outcome assessors, then transcribed into an electronic database and stored in it with password protection.

## Discussion

As one of the common forms of traditional Chinese exercise, Baduanjin has attracted increasing number of practitioners worldwide and considerable attention from the research community to determine the health benefits of Baduanjin exercise in both healthy and clinical populations. Compared with other aerobic exercises, Baduanjin is easy to learn with little physical demanding. And different from resistance training with the same level of intensity, Baduanjin does not have a series of soft and coherent movements to appeal practitioners to persevere, but also involves regulating mind and breathing to cultivate an internal energy [10]. Therefore, Baduanjin exercise can be implementable in clinical practice for older adults.

Since previous studies have demonstrated a link between sarcopenia and several adverse health outcomes like falls and fractures [1, 3], and Baduanjin exercise has advantages in improving balance, gait and physical function and reducing falls [14, 15, 25–28]. In order to provide another effective and safe exercise intervention for elderly people, who lack access or motivation to participate in a rigorous exercise program, to combat sarcopenia and minimize such adverse events, we really hope regular Baduanjin exercise can be a promising choice for them. For this reason, this trial was proposed to test whether older adults with sarcopenia can benefit from regular Baduanjin exercise. Several measures will be taken to control bias, including randomization, blinding of outcome assessors and statisticians, employing qualified physical exercise coaches. Physical and cognitive functions are concerned in this study, as well as composition and abundance of gut microbiota, because of the growing field of gut-muscle axis in neurology. These will provide valuable information to researchers, clinicians, and patients, and hold great promise in better understanding and treating older adults with sarcopenia.

We recognize that there are a few limitations in our study. As this is a single center trial, the results may not be generalizable to the wider population outside China. Additionally, the optimal intensity and timing of Baduanjin exercise remain unknown, but we believe that the Baduanjin exercise scheme used in the trial can provide information for researchers and clinicians to make decisions when they plan further studies on sarcopenia. Last but not the least, we may encounter challenges in recruiting and conducting, a higher dropout rate of clinical trials focused on chronic diseases may be inevitable due to the COVID-19 pandemic. To address these problems, we will encourage participants to practice the exercise at their home after they master the whole training program, and the form of live stream will also be taken if necessary.

Despite these limitations, this trial will determine if Baduanjin exercise is likely to be an effective non-pharmacological approach to treating sarcopenia and easily implementable for old patients with sarcopenia. Moreover, the results may also suggest its potential mechanism and encourage further studies on regular Baduanjin exercise to combat sarcopenia.

### Trial status

The study was registered with the Chinese Clinical Trial Register (ChiCTR2100051871) on October 8th, 2021, and modified on December 3rd, 2021. The subject recruiting was started in February, 2022.

## Supplementary Information

**Additional file 1.** SPIRIT checklist

## Data Availability

Anonymized data are available from the corresponding author on reasonable request.

## Abbreviations

AWGS: Asian Working Group for Sarcopenia
BE: Baduanjin exercise
BMI: Body mass index
HR: Heart rate
IQR: Interquartile range
ITT: Intention-to-treat
MoCA: Montreal cognitive assessment scale
NC: Non-sarcopenia control
PPS: Per protocol set
RT: Resistance training
SPPB: Short Physical Performance Battery
TCM: Traditional Chinese medicine
